# Differential Expression Profile of MicroRNAs during Differentiation of Cardiomyocytes Exposed to Polychlorinated Biphenyls

**DOI:** 10.3390/ijms131215955

**Published:** 2012-11-28

**Authors:** Chun Zhu, Zhang-Bin Yu, Jin-Gai Zhu, Xiao-Shan Hu, Yu-Lin Chen, Yu-Fang Qiu, Zheng-Feng Xu, Lin-Mei Qian, Shu-Ping Han

**Affiliations:** 1 State Key Laboratory of Reproductive Medicine, Department of Pediatrics, Nanjing Maternity and Child Health Care Hospital Affiliated to Nanjing Medical University, Nanjing 210029, China; E-Mails: zhifangxibao@163.com (C.Z.); yuzhangbin@126.com (Z.-B.Y.); zhujingai1983@163.com (J.-G.Z.); huxiaoshanshan@126.com (X.-S.H.); chenyulin@126.com (Y.-L.C.); qiuyufangnj@126.com (Y.-F.Q.); 2 State Key Laboratory of Reproductive Medicine, Center of Prenatal Diagnosis, Nanjing Maternity and Child Health Care Hospital Affiliated to Nanjing Medical University, Nanjing 210029, China; E-Mail: njxzf@126.com; 3 Department of Cardiology, the First Affiliated Hospital of Nanjing Medical University, Nanjing 210029, China

**Keywords:** PCB, miRNAs, cardiac myocytes, microarrays

## Abstract

Exposure to persistent environmental pollutants, such as polychlorinated biphenyls (PCBs), is a risk factor for the development of congenital heart defects. MicroRNAs (miRNAs) have been shown to be involved in cardiac development. The objective of this study was to investigate changes in miRNA expression profiles during the differentiation of cardiomyocytes exposed to PCBs. For that purpose, PCBs (Aroclor 1254) at a concentration of 2.5 μmol/L were added on day 0 of differentiation of P19 mouse embryonal carcinoma cells into cardiac myocytes. The relative expression of miRNA genes was determined by miRNA microarray and real-time reverse transcriptase polymerase chain reaction (real-time RT-PCR) analyses. The microarray results revealed that 45 miRNAs, of which 14 were upregulated and 31 were downregulated, were differentially expressed in P19 cells treated with PCBs compared with control cells. The miRNA expression data was validated with real-time RT-PCR. The expression of certain potential target genes (*Wnt1*) was found to be reduced in P19 cells treated with PCBs, whereas the expression of other potential predicted target genes (*GSK3*β) was increased. Our results demonstrate a critical role of miRNAs in mediating the effect of PCBs during the differentiation of P19 cells into cardiac myocytes.

## 1. Introduction

Polychlorinated biphenyls (PCBs), which belong to a class of chemicals called chlorinated hydrocarbons, are well-known environmental pollutants that have been widely used industrially and commercially for more than half a century [1,2]. PCB contamination is a serious environmental problem in China. For example, the total concentration of PCBs in some surface water and surface microlayers exceeds the limits set by the “Environmental Quality Standard for Surface Water” in the Jiangsu Province [3]. Numerous reports and studies have confirmed that PCB exposure leads to an increase in the incidence and severity of cardiovascular disease [4,5], but little is known about the underlying molecular mechanisms.

MicroRNAs (miRNAs) are an abundant class of small non-protein-coding RNAs that have been shown to play critical roles in a wide range of biological and pathological processes, including cardiac development and congenital heart defects [6]. However, the role of miRNAs in the differentiation of cardiomyocytes that have been exposed to PCBs has not been investigated thus far. The P19 mouse embryonal carcinoma cell line is a suitable model for studying cardiac differentiation at the molecular and functional levels. In the present study, we initially investigated the differential expression profiles of miRNAs during the differentiation of P19 cells exposed to Aroclor 1254. We used Aroclor 1254 because it is a well-known hepatotoxin and consists of a complex mixture of PCBs [7]. We subsequently confirmed the microarray results by real-time reverse transcription polymerase chain reaction (real-time RT-PCR). Furthermore, we investigated changes in the expression of the target genes of some miRNAs.

## 2. Results and Discussion

### 2.1. Assessment of Cell Differentiation

**Figure 1:**
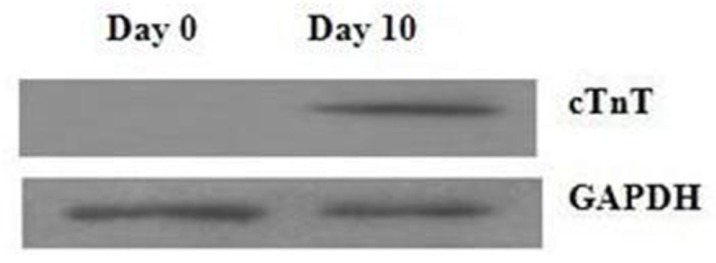
Assessment of cell differentiation. Expression of cTnT protein as shown by Western blotting. Abbreviations: D0, day 0; D10, day 10.

To investigate the differentiation of P19 cells into mature cardiomyocytes, we used Western blotting to identify the expression of cTnT protein (a myocyte differentiation marker). Figure 1 shows the increase in cTnT expression, indicating the differentiation of the cells into cardiac myocytes.

### 2.2. Morphology of P19 Cells during Differentiation

**Figure 2:**
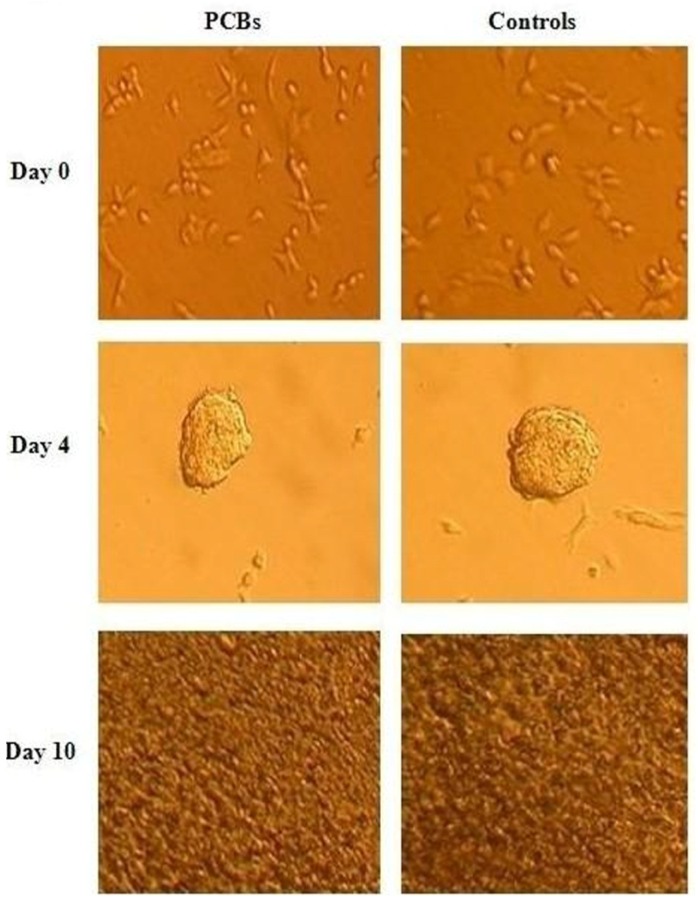
Morphology of P19 cells during differentiation into cardiac myocytes (day 0, 4, 10). No significant difference in morphology was observed between P19 cells exposed to PCBs and the control cells. Abbreviation: PCBs, polychlorinated biphenyls.

The changes in the morphology of P19 cells during differentiation were examined and photographed with an inverted microscope. P19 cells were allowed to aggregate from day 0 to day 4, and beating colonies were found on day 10. No significant differences in morphology were observed between P19 cells that had been exposed to PCBs and the control cells (Figure 2).

### 2.3. miRNA Expression Profiles in P19 Cells Exposed to PCBs Relative to Control Cells

**Figure 3:**
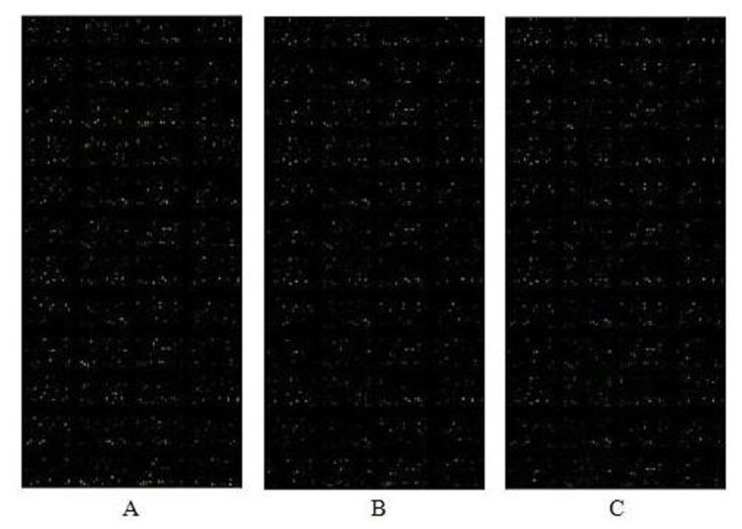
Detection of miRNAs by miRCURY LNA™ microarrays. The microarray slides contained three replicate subarrays, Abbreviation: miRNA, microRNA.

miRNA microarrays are a powerful tool for studying the biological function of miRNAs. Every sample was repeated three times to improve miRNA microarray accuracy (Figure 3). The miRNA expression pattern was found to be significantly different between cells that had been exposed to PCBs and controls (Table 1). A total of 45 miRNAs were differentially expressed in cardiomyocytes that differentiated from P19 cells exposed to PCBs (day 10) compared with normal cells (day 10). Specifically, 14 miRNAs were found to be upregulated and 31 miRNAs were downregulated in P19 cells exposed to PCBs.

### 2.4. Validation of Differentially Expressed miRNAs

**Figure 4:**
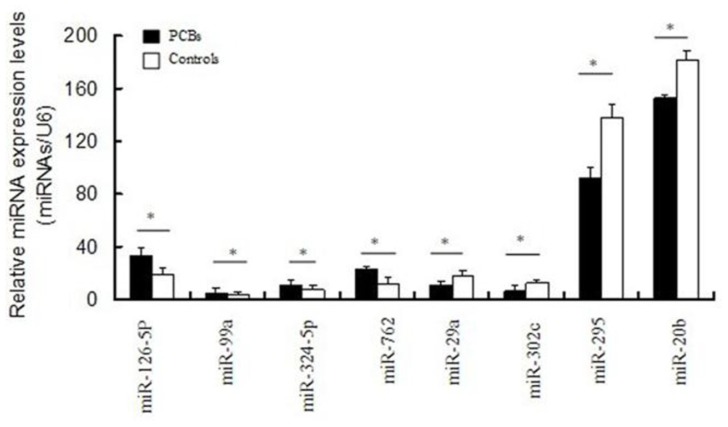
Validation of microarray data using real-time RT-PCR. The real-time RT-PCR reactions were repeated three times for every miRNA. Error bars indicate standard error. * *p* < 0.05. Abbreviations: miRNA, microRNA; PCBs, polychlorinated biphenyls; RT-PCR, reverse transcription polymerase chain reaction.

Eight miRNAs (miR-126-5p, miR-99a, miR-324-5p, miR-762, miR-29a, miR-302c, miR-295, miR-20b) were randomly selected to confirm the microarray results using real-time RT-PCR. The expression data obtained by real-time RT-PCR analysis were comparable to the results of the miRNA microarray analysis (Figure 4).

### 2.5. miRNA Target Predictions

**Figure 5:**
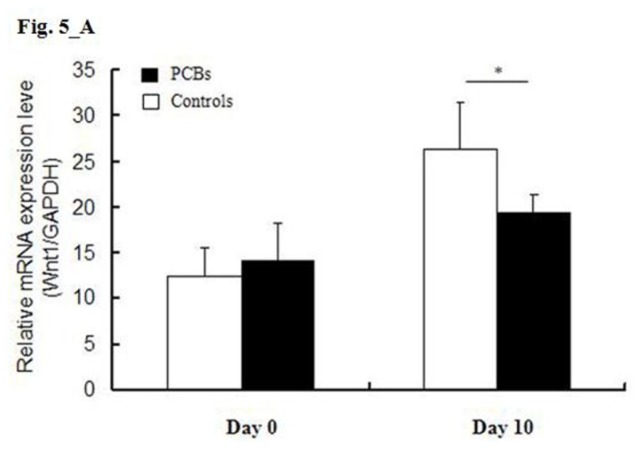

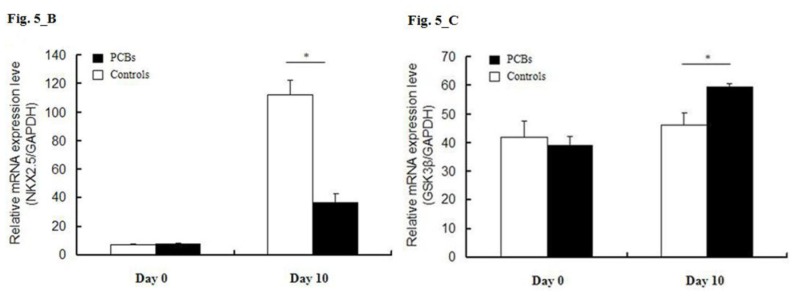
Expression of the miRNA targets as determined by real-time RT-PCR during the stimulation of differentiation (days 0 and 10). (**A**, **B**) The expression of *Wnt1* and *NKX2.5* was downregulated in P19 cells that were treated with PCBs, (**C**) whereas the expression of *GSK3*β was upregulated. * *p <* 0.05. Abbreviations: miRNA, microRNA; PCBs, polychlorinated biphenyls; RT-PCR, reverse transcription polymerase chain reaction.

In the course of exploring the targets of these miRNAs by computational prediction, we found that *Wnt1*, *GSK3*β, and *NKX2.5* were potential targets of miR-762, miR-29a, and miR-324-5p, respectively. The results of real-time RT-PCR analysis showed that the expression of *Wnt1* and *NKX2.5* was downregulated in P19 cells that were treated with PCBs, whereas the expression of *GSK3*β was upregulated (Figure 5).

### 2.6. Discussion

The heart is the first organ formed in the developing embryo. Exposure to PCBs, some of the most ubiquitous environmental contaminants, is a risk factor for the development of cardiovascular diseases. In this study, we simulated the exposure of PCBs with Aroclor 1254 at a concentration of 2.5 μmol/L, which is the concentration used in most experiments that examine the effect of PCBs on cells [2]. The P19 mouse embryonal carcinoma cell line can be specifically induced to differentiate into cardiac muscle cells, and is therefore one of the most suitable models for studying cardiac differentiation at the molecular and functional levels [8]. Thus, we selected the P19 cell line to investigate the differential expression profiles of miRNAs in the differentiation of P19 cells exposed to PCBs into cardiomyocytes.

In our initial expression profiling experiment we identified 45 miRNAs that were differentially expressed between P19 cells treated with PCBs and control cells. In particular, 14 miRNAs were upregulated and 31 were downregulated in P19 cells that had been exposed to PCBs during the differentiation process. To validate the microarray results, eight miRNAs were selected for further experimental confirmation: miR-126-5p, miR-99a, miR-324-5p, miR-762, miR-29a, miR-302c, miR-295, miR-20b. The expression levels of these miRNAs were measured with real-time RT-PCR, and the changes in the expression of all eight miRNAs were found to be consistent with the microarray data.

The role of miRNAs in cardiac development and cardiovascular disease has begun to be uncovered. MiR-208a has been previously shown to be necessary for normal cardiac conduction. The expression level of miR-208a has been demonstrated to gradually increase during mouse heart development. Transgenic overexpression of miR-208a was sufficient to induce hypertrophic growth in the mouse heart [9]. In the present study, miR-208a was upregulated in P19 cells exposed to PCBs, and could therefore have a role in the development of heart disease caused by PCB exposure. The results of a previous study suggest that miR-21 can protect against H_2_O_2_-induced injury via the induction of its target gene in cardiac myocytes [10]. Dong *et al.* also demonstrated the protective effect of miR-21 against ischemia-induced cardiac myocyte damage, an effect mediated by decreased cell apoptosis [11]. In our study, miR-21 was downregulated during differentiation in P19 cells exposed to PCBs. We speculate that PCBs might cause heart disease by downregulating the expression of miR-21. In the present study, the expression of *NKX2.5* was reduced in P19 cells treated with PCBs and *NKX2.5* is a potential target of miR-324-5p. *NKX2.5*, a member of the NK2 class homeodomain protein, is one of the earliest genes expressed in vertebrate heart development. *Nkx2.5* plays an important role in the development of heart tube, ventricular septal and endocardial cushion [12,13]. This suggests that miR-324-5p might be important in controlling pathological events in congenital heart disease caused by exposure to PCBs.

Numerous reports and studies show that the Wnt signaling pathway has a key role in the process of cardiovascular differentiation and morphogenesis [14,15]. At the cell surface, Wnt proteins activate the pathway through binding a receptor complex containing Frizzled, whereas GSK3β negatively regulates the pathway [16]. During the differentiation of P19 cells treated with PCBs, we found that increased expression of miR-762 was associated with a decrease in the expression of *Wnt1* and an increase in *GSK3*β expression. These findings indicate that miRNAs usually cause translational repression or target degradation and gene silencing [17]. The function of these miRNAs, and the relationship between miRNAs and their targets, requires further confirmation and study.

## 3. Experimental Methods

### 3.1. Cell Culture

The P19 mouse embryonal carcinoma cells used in this study were obtained from the American Type Culture Collection (ATCC, Manassas, VA, USA). In order to induce cardiac differentiation, the cells were cultured in α-MEM (Gibco BRL, Grand Island, NY, USA) containing 10% fetal bovine serum (FBS; Gibco BRL), 100 U/mL penicillin, and 100 μg/mL streptomycin at 37 °C in 5% CO_2_ until confluence. After 48 h, at which point the cells had reached confluence (day 0), the cells were cultured in α-MEM, 1% DMSO (Sigma, St. Louis, MO, USA), 10% FBS, 100 U/mL penicillin, and 100 μg/mL streptomycin for 96 h (day 4) in bacteriological dishes. The cell aggregates were transferred to 6 cm bacterial dishes, and cultured in α-MEM containing 10% FBS for an additional 4 or 6 days (day 10) until the cells differentiated into beating cardiomyocytes. The morphological changes of the cells were examined and photographed with an inverted microscope (Nikon, Japan).

Solutions of Aroclor 1254 at a concentration of 2.5 μmol/L were prepared in DMSO. The Aroclor 1254 was added into the medium on day 0 of differentiation. We chose this concentration because it has been used in a previous study [2]. The same amount of DMSO diluted with the culture medium was used as a control.

### 3.2. Western Blotting

Treated cells were washed with ice-cold PBS and lysis buffer (50 mmol/L Tris-HCl, 1% Triton X-100, 0.2% sodium deoxycholate, 0.2% SDS, and 1 mmol/L EDTA at pH 7.4) and vortexed briefly. The lysate supernatant was collected after centrifugation at 15,200× *g* at 4 °C for 15 min. Protein levels were quantified using a protein assay reagent kit, and quantification was performed as described previously [18]. Western blotting was carried out using a monoclonal rabbit anti-cTnT antibody (Chemicon, Temecula, CA, USA) and a monoclonal rabbit anti-GAPDH antibody (Proteintech Group, Inc., Wuhan, China).

### 3.3. Total RNA Preparation

Total RNA was extracted from cardiac myocytes (on day 10) that had been exposed to either Aroclor 1254 or the control solution. Total RNA was extracted by using Trizol (Invitrogen, Carlsbad, CA, USA) according to the manufacturer’s instructions. Total RNA was quantified by a nanodrop spectrophotometer (ND-1000; NanoDrop Technologies, Wilmington, DE, USA).

### 3.4. MiRNA Microarray

Microarrays were performed by utilizing the miRCURY LNA™ microRNA Array (Version 14.0 Exiqon, Vedbaek, Denmark). All procedures were carried out according to the manufacturer’s instructions. After having passed RNA measurement on the NanoDrop instrument, the samples were labeled using the miRCURY™ Hy5™/Hy3™ Power labeling kit (Exiqon) and hybridized on the miRCURY LNA™ Array (v.14.0), which contains more than 1700 capture probes covering all miRNAs listed in miRBase version 14.0. After stopping the labeling procedure, the Hy3™-labeled samples and a Hy5™-labeled reference RNA sample were mixed pairwise and hybridized to the miRCURY LNA™ Array version 14.0 (Exiqon). The hybridization and subsequent wash steps were performed according to the miRCURY LNA™ array manual. The microarray slides were scanned using the ScanArray™ 4000 XL scanner (Packard Biochip Technologies, Billerica, MA, USA) and the image analysis was carried out using the ImaGeneTM 6.1.0 software (BioDiscovery, Inc., El Segundo, CA, USA). The normalized data was analyzed using the locally weighted scatter plot smoothing (lowess) regression algorithm (MIDAS, TIGR Microarray Data Analysis System). Statistical comparisons were performed by using the analysis of variance (ANOVA) statistic.

### 3.5. Validation of Microarray Experiments

Real-time RT-PCR analysis was performed to validate the miRNA microarray results. Total RNA was isolated from cultured cells with Trizol (Invitrogen, Carlsbad, CA, USA). The reverse transcription mixture contained 1 μg total RNA, 0.5 μL miRNA reverse primer (1 μM) (Table 2), 0.3 μL RNase inhibitor (40 U/μL), 2 μL 10× buffer, 2 μL RNasin (10 U/μL), and an appropriate amount of RNAse-free H_2_O to a total volume of 20 μL. The reverse transcription reaction was performed at 16 °C for 30 min, then 42 °C for 40 min, followed by heat inactivation at 85 °C for 5 min. Real-time PCR analysis was performed on an ABI 7300 real-time PCR system (Applied Biosystems, Carlsbad, CA, USA) with a 25 μL volume reaction containing 2.5 μL reverse transcription product, 0.5 μL forward and reverse primer (Table 3), and 12.5 μL SYBR-Green real-time PCR master mix. The reactions were incubated in 96-well plates at 95 °C for 5min, followed by 40 cycles (95 °C for 10 s, 60 °C for 20 s, 72 °C for 20 s, 78 °C for 20 s), and then ramped from 72 °C to 99 °C to obtain the melting curve. The *U6* expression level was used as an internal control for miRNA expression levels. All reactions were run at least in triplicate.

### 3.6. MiRNA Target Predictions and Real-Time RT-PCR

Predicted targets of miR-762, miR-29a, and miR-324-5p were analyzed in the Sanger Center miRNA registry [19], TargetScan and PicTar target predictions [20]. The expression of the target genes was measured using real-time RT-PCR. Briefly, RNA extraction, purification, and reverse transcription were performed as stated above. Real-time RT-PCR using the universal TaqMan probe was performed and the results were analyzed using a 7300 Real-Time PCR system under the following conditions: samples were incubated at 95 °C for 10 min for initial denaturation, followed by 40 cycles of amplification that were performed at 95 °C for 15 s and at 60 °C for 1 min. A modification of the 2^ΔΔ^*^C^*^t^ method was used to analyze the relative quantification data [21]. *GAPDH* was used as the internal control gene. The sequences of the primers used are shown in Table 4.

## 4. Conclusion

In summary, we identified miRNAs that are differentially expressed during the differentiation of cardiomyocytes from P19 cells exposed to PCBs and control cells. Our results suggest that specific miRNAs could be directly involved in the development of cardiovascular disease resulting from exposure to PCBs. The function of these miRNAs will be analyzed in depth in the future.

## Figures and Tables

**Table 1:** miRNAs differentially expressed between cardiomyocytes that differentiated from P19 cells exposed to PCBs (day 10) compared with normal cells (day 10).

miRNA	Regulation	Log2	*p*-Value
mmu-miR-99a	Up	1.74	0.0060
mmu-miR-214^*^	Up	1.51	0.0365
mmu-miR-345-5p	Up	1.57	0.0128
mmu-miR-126-5p	Up	2.89	0.0338
mmu-miR-762	Up	2.20	0.0242
mmu-miR-324-5p	Up	1.90	0.0071
mmu-miR-181a	Up	1.76	0.0318
mmu-miR-302b^*^	Up	2.83	0.0404
mmu-miR-155	Up	1.97	0.0333
mmu-miR-33	Up	1.90	0.0487
mmu-miR-208a	Up	2.13	0.0465
mmu-miR-10a	Up	1.84	0.0226
mmu-miR-154	Up	1.64	0.0392
mmu-miR-500	Up	1.53	0.0079
mmu-miR-29a	Down	0.60	0.0139
mmu-miR-302c	Down	0.65	0.0093
mmu-miR-302b	Down	0.73	0.0249
mmu-miR-293	Down	0.45	0.0349
mmu-miR-294	Down	0.52	0.0284
mmu-miR-295	Down	0.56	0.0410
mmu-miR-7a	Down	0.62	0.0435
mmu-miR-302a^*^	Down	0.67	0.0203
mmu-miR-1949	Down	0.58	0.0337
mmu-miR-21	Down	0.63	0.0039
mmu-miR-494	Down	0.54	0.0165
mmu-miR-20b	Down	0.55	0.0349
mmu-miR-200b	Down	0.14	0.0021
mmu-miR-136	Down	0.37	0.0346
mmu-miR-141	Down	0.05	0.0249
mmu-miR-194	Down	0.02	0.0147
mmu-miR-29b	Down	0.17	0.0350
mmu-miR-32	Down	0.48	0.0075
mmu-miR-710	Down	0.29	0.0120
mmu-miR-290-3p	Down	0.35	0.0204
mmu-miR-2133	Down	0.45	0.0072
mmu-miR-18b	Down	0.61	0.0213
mmu-miR-101a	Down	0.65	0.0273
mmu-miR-326	Down	0.47	0.0095
mmu-miR-669c	Down	0.35	0.0428
mmu-miR-302d	Down	0.58	0.0113
mmu-miR-2141	Down	0.25	0.0286
mmu-miR-183	Down	0.60	0.0151
mmu-miR-363	Down	0.56	0.0174
mmu-miR-706	Down	0.64	0.0216
mmu-miR-290-3p	Down	0.34	0.0439

**Table 2:** Real time (RT) primer sequences.

Gene name	RT primer
mmu-miR-126-5p	5′ GTCGTATCCAGTGCGTGTCGTGGAGTC
	GGCAATTGCACTGGATACGACCGCGTA 3′
mmu-miR-99a	5′ GTCGTATCCAGTGCGTGTCGTGGAGTC
	GGCAATTGCACTGGATACGACCACAAG 3′
mmu-miR-324-5p	5′ GTCGTATCCAGTGCGTGTCGTGGAGTC
	GGCAATTGCACTGGATACGACACACCA 3′
mmu-miR-762	5′ GTCGTATCCAGTGCGTGTCGTGGAGTC
	GGCAATTGCACTGGATACGACGCTCTG 3′
mmu-miR-29a	5′ GTCGTATCCAGTGCGTGTCGTGGAGTC
	GGCAATTGCACTGGATACGACTAACCG 3′
mmu-miR-302c	5′ GTCGTATCCAGTGCGTGTCGTGGAGTC
	GGCAATTGCACTGGATACGACCCACTG 3′
mmu-miR-295	5′ GTCGTATCCAGTGCGTGTCGTGGAGTC
	GGCAATTGCACTGGATACGACAGACTCAA 3′
mmu-miR-20b	5′ GTCGTATCCAGTGCGTGTCGTGGAGTC
	GGCAATTGCACTGGATACGACCTACCT 3′
U6	5′ CGCTTCACGAATTTGCGTGTCAT 3′

**Table 3:** Primers for real-time RT-PCR.

Gene name	Primers	Product size (bp)
mmu-miR-126-5p	F: 5′ GGGGCATTATTACTTTTGG 3′	65
	R: 5′ CAGTGCGTGTCGTGGA 3′	
mmu-miR-99a	F: 5′ GATAACCCGTAGATCCGAT 3′	65
	R: 5′ CAGTGCGTGTCGTGGA 3′	
mmu-miR-324-5p	F: 5′ CGCATCCCCTAGGGCA 3′	61
	R: 5′ GTGCGTGTCGTGGAGTCG 3′	
mmu-miR-762	F: 5′ ATGCTGGGGCCGGGA 3′	59
	R: 5′ GTGCGTGTCGTGGAGTCG 3′	
mmu-miR-29a	F: 5′ GGGTAGCACCATCTGAAAT 3′	62
	R: 5′ TGCGTGTCGTGGAGTC 3′	
mmu-miR-302c	F: 5′ GGCAAGTGCTTCCATGTTT 3′	65
	R: 5′ CAGTGCGTGTCGTGGAGT 3′	
mmu-miR-295	F: 5′ GGGGGAAAGTGCTACTACTT 3′	68
	R: 5′ CAGTGCGTGTCGTGGAG 3′	
mmu-miR-20b	F: 5′ GGACAAAGTGCTCATAGTGC 3′	66
	R: 5′ CAGTGCGTGTCGTGGAGT 3′	
U6	F: 5′ GCTTCGGCAGCACATATACTAAAAT 3′	89
	R: 5′ CGCTTCACGAATTTGCGTGTCAT 3′	

**Table 4:** Primers used for miRNA target prediction.

Gene name	Primers	Product size (bp)
*Wnt1*	F: 5′ GGTTTCTACTACGTTGCTACTGG 3′	121
	R: 5′ GGAATCCGTCAACAGGTTCGT 3′	
*GSK3β*	F: 5′ TGGCAGCAAGGTAACCACAG 3′	189
	R: 5′ CGGTTCTTAAATCGCTTGTCCTG 3′	
*NKX2.5*	F: 5′ CCTGCGGCCTCTACATGA 3′	222
	R: 5′ AGGGTCTCACCAGCAGGA 3′	
*GAPDH*	F: 5′ TTCACCACCATGGAGAAGGC 3′	237
	R: 5′ GGCATGGACTGTGGTCATGA 3′	
